# Eye movements dataset for objective-based assessment of object-oriented programming knowledge

**DOI:** 10.1016/j.dib.2023.109558

**Published:** 2023-09-09

**Authors:** Chandrika K R, Amudha J

**Affiliations:** Department of Computer Science and Engineering, Amrita School of Computing, Amrita Vishwa Vidyapeetham, Bangalore, India

**Keywords:** Eye-tracking, Programming, Assessment, MCQ, Dataset, Human-computer interaction, Eye movements and cognition, Empirical studies in HCI

## Abstract

The Eye Movements dataset for Objective Assessment contains eye gaze data and learners' scores in an objective-based object-oriented programming assessment. The learners’ knowledge was assessed for various programming concepts like object initialisation, variable declaration, constructors etc. The eye gaze data of learners were collected using the commercial eye tracker, and their responses using the Think Aloud method. The eye gaze data was then mapped to the contextual information in the stimulus, which included questions, keywords, and answer options. The raw and analysed data are available to learn learners' cognitive behavior during an objective assessment.

Specifications Table

**Table:** 

Subject	Computer Science Education
Specific subject area	Eye-tracking Data for Learner Assessment in Object-Oriented Programming
Type of data	Table
How the data were acquired	Data was gathered by eye-tracking using an SMI Redn 60 Hz Professional eye tracker device.
Data format	Raw Data Processed Data
Description of data collection	The experimental study is conducted in the eye-tracking research lab in the Department of Computer Science and Engineering, Amrita School of Computing, with 15 learners. All learners have prior programming knowledge and are undergoing an introductory course in Java Programming. The experiment was designed in the SMI Experimental Suite 360֯. The remote eye-tracking device was mounted on the laptop at a distance of 60–70 cm. The responses of the learners were collected using the Think Aloud method.
Data source location	· Institution: Amrita Vishwa Vidyapeetham · City/Town/Region: Bengaluru · Country: India
Data accessibility	Repository name: Eye movements dataset for objective-based assessment of object-oriented programming knowledge Data identification number: 10.17632/795 gmyst22.3 Direct URL to data: https://data.mendeley.com/datasets/795gmyst22/3
Related research article	Ramachandra, C.K.; Joseph, A. IEyeGASE: An Intelligent Eye Gaze-Based Assessment System for Deeper Insights into Learner Performance. Sensors 2021, 21, 6783. https://doi.org/10.3390/s21206783

## 1 Value of the Data

The Eye Movements dataset for Objective Assessment (EMOA) consists of learners’ demographic information and eye gaze data collected for an empirical study to understand their cognitive behavior during an objective-based assessment in an object-oriented programming language.

• The dataset can be used to infer the learner’s problem-solving process and cognitive traits.
• The task designed in the empirical study can be used as a template to construct new tasks to analyze learners’ problem-solving processes at different levels of thinking as described by Bloom’s Taxonomy.
• The design can also be extended to conduct similar empirical studies to understand learner knowledge in different courses.

## 2 Objective

The EMOA dataset was collected as no dataset is available in the literature to understand learners' problem-solving processes and cognitive traits during the objective assessment for programming language. The objective assessment was based on the lower level of Bloom's Taxonomy [1] called Remembrance on topics like keywords, operators, decision-making, loops, constructors, object creation, and initialization of object-oriented programming paradigm. The dataset was analyzed to get insights into cognitive traits like confidence level, quickness in task completion, task engagement, inattentional blindness to critical information, and wavering behavior [2]. The dataset is available for researchers to analyze various aspects of learners in an objective-based assessment. It can also be used in creating machine learning models to predict learner performance and behavior on similar tasks.

## 3 Data Description

The EMOA dataset consists of three files- EMOADemographics.csv, EMOAEventData.csv, and EMOASemanticData.csv. The EMOADemographics.csv consists of the learner's information about age, gender, prior knowledge in programming, and level of education pursued. EMOAEventData.csv contains learners' event data like total fixation, saccade, and blink data generated using SMI Redn Professional BeGaze Software. Table 1 represents the participants’ demographic information, and Table 2 represents the event data description.

**Table 1:** Demographic information of participants as in EMOADemographics.csv.

Field	Description
Participant Code	Participant Identification. Identified as STUD-P01…….STUD-P15
Gender	{Male, Female}
Age	Age in years {18,19}
Prior Programming Knowledge	{Yes, No}
Education Qualification	{Graduate, Post Graduate}

**Table 2:** Event Data Description of EMEventData.csv.

Field	Description
Trial	The trial number identified as Trial001, Trial002,… Trial 005
Stimulus	Stimulus Identifier Identified as MCQ1, MCQ2….MCQ5
Participant	Participant Identification. Identified as STUD-P01…….STUD-P15
Fixation Count	Fixations in a Trial
Fixation Duration Total [ms]	Duration of Fixation for the Trial in ms
Saccade Count	Number of Saccades in the Trial
Saccade Duration	Saccade Duration in the Trial in ms
Saccade Amplitude Total [°]	Saccade Amplitude in degrees
Blink Count	Number of blinks in the Trial
Scanpath Length[px]	Scanpath length in the Trial in pixels

**Fig. 1:**
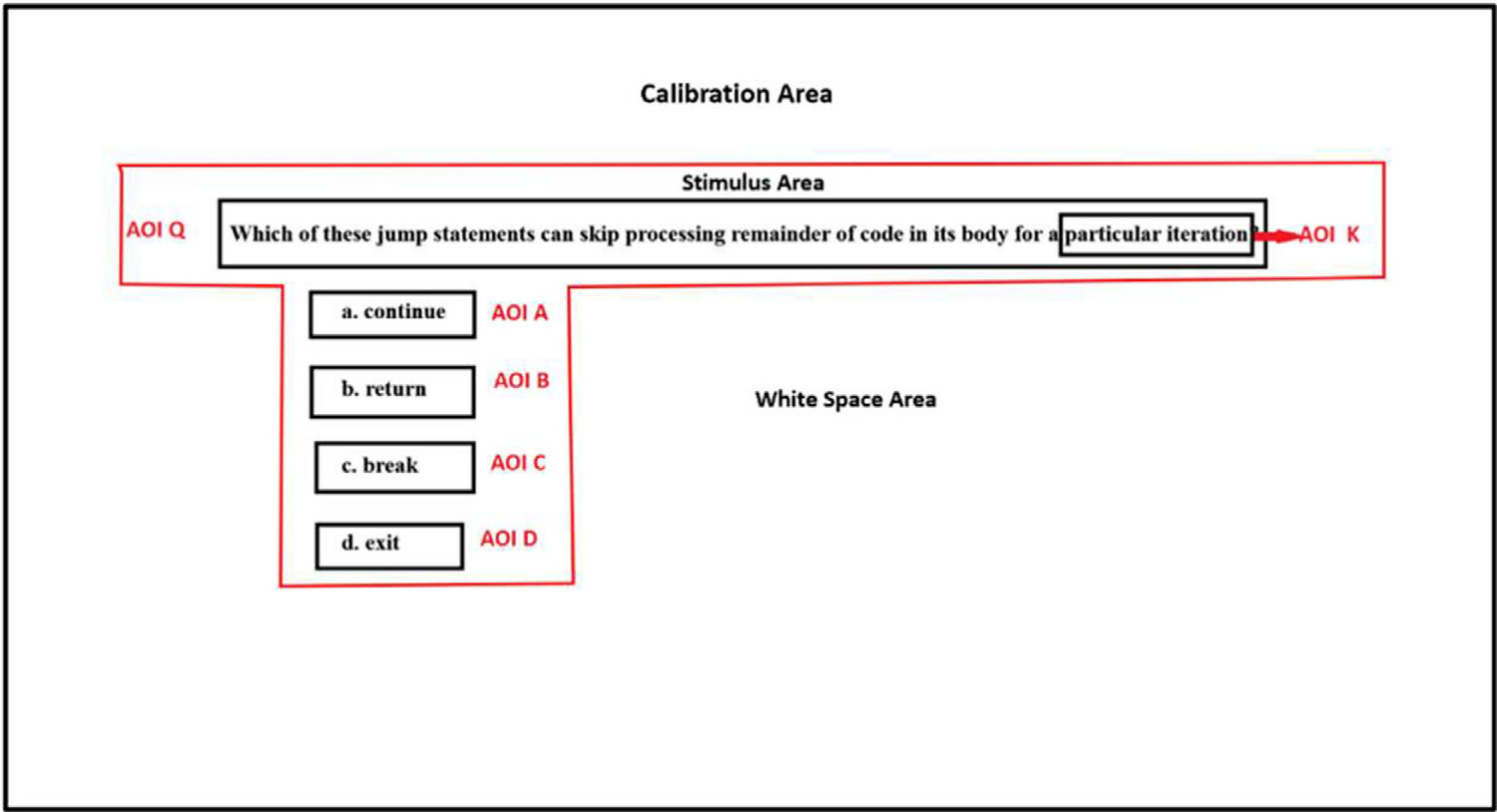
Area of Interest information of MCQ 1.

The file EMOASemanticData.csv consists of the semantic gaze information of learners on various Area of Interest(AOI). Each Multiple Choice Question (MCQs) comprises six AOI regions- Question, Option A, Option B, Option C, Option D and Keyword, as shown in Fig. 1. The Question is the MCQ question; Option A, Option B, Option C and Option D are the various choices in the MCQ. Keyword is the key information in the question that helps the learner identify the right option in the MCQ. For each of these AOIs fixation duration and fixation count were computed using the open gaze analysis platform PyGaze [3]. These gaze information are called semantic features as they provide contextual information of what is viewed by the learner. Table 3 consists of the semantic features.

**Table 3:** Semantic Features as in EMOASemanticData.csv.

Field	Description
Participant Code	Participant Identification. Identified as STUD-P01…….STUD-P15
Trial	Trial Information. Identified as Trial001, Trial002,… Trial 005
Correctly Answered	Response of the learner was correct(Yes) or incorrect (No)
Total Fixation	Number of Fixations in the Trial
Total Duration	Total Fixation Duration for the Trial in ms
Q fix	Question's Fixations
Q dur	Question's Duration of Fixation
A fix	Option A's Fixations
A dur	Option A's Duration of Fixation
B fix	Option B's Fixations
B dur	Option B's Duration of Fixation
C fix	Option C's Fixations
C dur	Option C's Duration of Fixation
D fix	Option D's Fixations
D dur	Option D's Duration of Fixation
K fix	Keyword's Fixations
K dur	Keyword's Duration of Fixation

## 4 Experimental Design, Materials and Methods

The experimental design consists of the setup, stimulus design and participant recruitment.

### 4.1 Experimental setup

The experimental setup comprises hardware and software for data collection. The hardware system consists of a laptop, and a remote-mounted eye tracker, SMI Redn Professional, with 60 Hz [4]. The eye tracker was mounted on the lower part of the laptop screen 60–70 cm away from the learner. The experiment was set up using SMI Experimental Suite 360° [5]. The dataset was processed and exported from SMI BeGaze Software [6]. The EMTADataset was further analyzed and the semantic features were then generated using in-house algorithms running on PyGaze platform.

### 4.2 Stimulus design

Five MCQs based on various object-oriented concepts were given as an assessment task. Each MCQ has a question, a keyword and four options. The keyword is the critical information in the question. All the MCQs were designed to assess the learners at a lower level of Bloom's Taxonomy called Remembrance. The following are the details of MCQs, the related concept, the keywords that provide valuable information towards the correct choice and various answer options as in the experimental study [2]:

• MCQ1: Which keyword is used by method to refer to the object that invoked it?

The Keyword: object that invoked it

Options

1. import (Incorrect)
2. this (Correct)
3. catch (Incorrect)
4. super (Incorrect)

Concept: Object creation and initialization

• MCQ2: List the arithmetic operators in increasing order of precedence.

The Keyword: increasing order of precedence

Options

1. * +% - / (Incorrect)
2. * / - +% (Incorrect)
3. * /% + - (Correct)
4. *% / + - (Incorrect)

Concept: Operators in Java

• MCQ3: Which of the following are keywords in Java?

The Keyword: keywords

Options

1. while, switch, if, static, bool (Incorrect)
2. while, switch, Boolean, static, pack(Incorrect)
3. break, catch, ball, return, switch (Incorrect)
4. while, switch, break, Boolean, catch (Correct)

Concept: Keywords in Java

• MCQ4: Which keyword is used to invoke base class constructor?

The Keyword: invoke base class constructor

Options

1. this (Incorrect)
2. import (Incorrect)
3. refer (Incorrect)
4. super (Correct)

Concept: Constructor

• MCQ5: Which of these jump statements can skip processing remainder of code in its body for a particular iteration?

The Keyword: particular iteration

Options

1. continue (Correct)
2. return (Incorrect)
3. break (Incorrect)
4. exit (Incorrect)

Concept: Decision-Making and Loops

### 4.3 Participants

The participants were fifteen learners in their second year of graduation in the Department of Computer Science and Engineering. The eye-tracking studies in computer programming education [7] shows that the number of participants usually vary from 2 to 82. The selection of participants are related directly to the aim of the study. All the learners were in the age group of 18–19 years. There were three female and twelve male learners. The learners who volunteered for the study had prior knowledge of programming. Written consent was signed by all the learners and no rewards were given to them.

### 4.4 Experiment procedure

The experimental study was conducted in the eye tracking lab at Amrit School of Computing, Amrita Vishwa Vidyapeetham, Bangalore. Separate time slots for one hour were scheduled for each learner. The learners were briefed on the study's objective and the do's and don'ts. Before the study, a nine-point calibration was conducted to obtain a functional mapping of eye tracker output to the gaze point. The learners were then presented with the MCQs in the same order. There was no restriction on the time for answering the MCQs. The Think Aloud method recorded the learners' responses [8]. In think aloud method, the learner thinks aloud the answer(or response) that the observer of the experiment manually records.

## Ethics

Informed consent was obtained from the learners who participated in the study. Study was performed with ethical clearance from the Ethics Committee (non-medical) at the Amrita Vishwa Vidyapeetham, clearance number: AMRITA/SOE/ADMN/DOE/10/2021/01.

## CRediT authorship contribution statement

**Chandrika K R:** Conceptualization, Methodology, Data curation, Writing – original draft. **Amudha J:** Supervision, Validation, Writing – review & editing.

## Data Availability

Eye-tracking Data for Learner Assessment in Object-Oriented Programming (Original data) (Mendeley Data).
